# The Heme Oxygenase System Suppresses Perirenal Visceral Adiposity, Abates Renal Inflammation and Ameliorates Diabetic Nephropathy in Zucker Diabetic Fatty Rats

**DOI:** 10.1371/journal.pone.0087936

**Published:** 2014-01-30

**Authors:** Joseph Fomusi Ndisang, Ashok Jadhav, Manish Mishra

**Affiliations:** Department of Physiology, University of Saskatchewan College of Medicine, Saskatoon, Saskatchewan, Canada; University of Tokushima, Japan

## Abstract

The growing incidence of chronic kidney disease remains a global health problem. Obesity is a major risk factor for type-2 diabetes and renal impairment. Perirenal adiposity, by virtue of its anatomical proximity to the kidneys may cause kidney disease through paracrine mechanisms that include increased production of inflammatory cytokines. Although heme-oxygenase (HO) is cytoprotective, its effects on perirenal adiposity and diabetic nephropathy in Zucker-diabetic fatty rats (ZDFs) remains largely unclear. Upregulating the HO-system with hemin normalised glycemia, reduced perirenal adiposity and suppressed several pro-inflammatory/oxidative mediators in perirenal fat including macrophage-inflammatory-protein-1α (MIP-1α), endothelin (ET-1), 8-isoprostane, TNF-α, IL-6 and IL-1β. Furthermore, hemin reduced ED1, a marker of pro-inflammatory macrophage-M1-phenotype, but interestingly, enhanced markers associated with anti-inflammatory M2-phenotype such as ED2, CD206 and IL-10, suggesting that hemin selectively modulates macrophage polarization towards the anti-inflammatory M2-phenotype. These effects were accompanied by increased adiponectin, HO-1, HO-activity, atrial-natriuretic peptide (ANP), and its surrogate marker, urinary-cGMP. Furthermore, hemin reduced renal histological lesions and abated pro-fibrotic/extracellular-matrix proteins like collagen and fibronectin that deplete nephrin, an important transmembrane protein which forms the scaffolding of the podocyte slit-diaphragm allowing ions to filter but not massive excretion of proteins, hence proteinuria. Correspondingly, hemin increased nephrin expression in ZDFs, reduced markers of renal damage including, albuminuria/proteinuria, but increased creatinine-clearance, suggesting improved renal function. Conversely, the HO-blocker, stannous-mesoporphyrin nullified the hemin effects, aggravating glucose metabolism, and exacerbating renal injury and function. The hemin effects were less-pronounced in Zucker-lean controls with healthy status, suggesting greater selectivity of HO in ZDFs with disease. We conclude that the concomitant reduction of pro-inflammatory/oxidative mediators, macrophage infiltration and profibrotic/extracellular-matrix proteins, coupled to increased nephrin, adiponectin, ANP, cGMP and creatinine clearance may account for improved renal function in hemin-treated ZDFs. These findings suggest that HO-inducers like hemin may be explored against the co-morbidity of perirenal adiposity and diabetic nephropathy.

## Introduction

Recent epidemiological data indicates that more than 1.6 billion adults worldwide are overweight and over 400 million are obese [1], [2]. Obesity is a major risk factor for insulin-resistant type-2 diabetes mellitus (T2D), dyslipidemia, hypertension and impaired renal function [3]–[6]. One of the common causes of morbidity and mortality in T1D and T2D patients is diabetic nephropathy, a micro-vascular complication of diabetes that may lead to end-stage-renal-disease (ESRD) [7]. The growing incidence of chronic kidney disease is widely recognized as a global health problem. The prevalence and incidence of ESRD is greater in patients co-morbid with obesity and diabetes [8]. Moreover, perirenal adiposity is an independent predictor of kidney dysfunction in T2D [9]. Thus, novel strategies that could simultaneously combat obesity, insulin resistant T2D and diabetic nephropathy are needed.

It is widely acknowledged that the site of fat accumulation may be more critical for health than the overall amount of fat tissue [10]. Moreover, adipocytes from different body compartments have distinct inflammatory phenotype based on their anatomical location [10]. Generally, visceral or intra-abdominal adiposity is more-malignant than subcutaneous adiposity, although they are both implicated in the pathogenesis of obesity-related cardio-metabolic complications like insulin resistance, T2D and renal disease [10], [11]. Perirenal adiposity, in comparison to central obesity is a greater risk factor for renal complications [9]. Emerging evidence indicates that perirenal adiposity may better reflect the risks commonly associated with increased visceral fat accumulation and particularly those related to impaired renal function [9]. By virtue of its anatomical and functional proximity to the kidney, perirenal adiposity may be even more malignant than central adiposity. Perirenal adiposity can lead to renal impairment through paracrine mechanisms that include increased production of inflammatory cytokines including tumour necrosis factor alpha (TNF-α), interleukin (IL)-6 and IL-1β and interestingly, these cytokine are also implicated in dysfunctional glucose metabolism [12]–[16]. Moreover, increased perirenal adiposity has been shown to compress renal vessels and renal parenchyma, causing elevated renal interstitial hydrostatic fluid with reduction of renal and tubular flow rates [17]. Therefore, novel formulations capable of reducing perirenal adiposity and its deleterious cytokines are needed to safeguard renal morphology and function.

In diabetic nephropathy, the expression of nephrin is deregulated [18], and elevated levels of pro-fibrotic/extracellular matrix proteins such as collagen and fibronectin are implicated in the aberrant expression of nephrin [19]. Nephrin is an important transmembrane zipper-like protein which is critical for the formation of the scaffolding of the podocyte slit diaphragm of the glomerular barrier, a structure that regulates the aperture size of the renal filtration barrier, selectively allowing the filtration of small molecules like ions, but not larger molecules like proteins [20]–[22]. A defect in nephrin may cause massive excretion of proteins, hence proteinuria [20]–[22]. Therefore, agents capable of reducing excessive deposition of pro-fibrotic/extracellular matrix proteins may be useful to preserve nephrin, and thus improve renal dysfunction due to proteinuria.

Although we recently reported the insulin sensitizing and cytoprotective effects of the heme oxygenase (HO) inducer, hemin, in Zucker diabetic Fatty rats (ZDFs) [23], [24], the effects of the HO system on perirenal adiposity remains largely unclear. Similarly, the effects of upregulating the HO system with hemin on macrophage polarization in renal tissue have not been reported. The two common polarized macrophage phenotypes are the pro-inflammatory M1-phenotype and anti-inflammatory M2-phenotype, and these subtypes are often referred to as classically activated macrophages (M1 macrophages) and alternatively activated macrophages (M2 macrophages) 13,25,26. Importantly, we will investigate whether hemin therapy can selectively modulate M1 and M2 macrophages in the kidneys to counteract inflammatory insults. Whether the effects of hemin therapy on M2 macrophage will be accompanied by increased expression of the anti-inflammatory cytokine, IL-10 [27] will also be investigated. Given that macrophage-inflammatory-protein-1α (MIP-1α) is a chemokine implicated in macrophage infiltration [28], we will also assess the effect of hemin therapy on this protein.

Therefore this study will unveil the effects of hemin therapy on renal expression of the anti-inflammatory macrophage M2-phenotye, the pro-inflammatory macrophage M1-phenotype and related chemokines/cytokines including MIP-1α, TNF-α, IL-6 and IL-1β as well as nephrin and pro-fibrotic/extracellular matrix proteins such as collagen and fibronectin. Importantly, the effects of the HO-system on perirenal adiposity, MIP-1α, M1/M2 macrophage and nephrin in ZDFs have not been reported. Since the mechanisms by which hemin therapy improves renal function in the co-morbid conditions of obesity and insulin-resistant diabetes have not been completely characterized, this study will unmask novel effects of hemin on perirenal adiposity and diabetic nephropathy in ZDFs, and add more insights in the multifaceted complication of diabetic nephropathy.

## Materials and Methods

### Animals Groups and Plasma Measurements

The experimental protocol was approved by University of Saskatchewan Standing Committee on Animal Care and Research Ethics. Male ZDF of 12 weeks and age/sex-matched litter Zucker lean (ZL) rats were purchased from Charles River (Willington, MA, USA). The animals were housed at 21°C with 12-hour light/dark cycles, fed with fed with Purina 5008 diet and had access to drinking water *ad libitum*. After a week of acclimation, the animals were randomly assigned to the following experimental groups: **(A)** controls (ZDF and ZL), **(B)** hemin-treated ZDF, **(C)** hemin-treated ZL, (**D**) ZDF treated with hemin and the HO inhibitor, stannous mesoporphyrin (SnMP), (**F**) ZDF treated with SnMP alone, and **(G)** ZDF and ZL treated with vehicle dissolving hemin and SnMP. Hemin (15 mg/kg i.p.) and SnMP (5 mg/100 g body weight, ip) were prepared and administered twice weekly for a duration of 8 weeks as we previously described [24], [29], [30].

Fasting glucose was monitored weekly with a diagnostic auto-analyzer (BD, Franklin Lakes, NJ) after 6hrs of fasting as e previously reported [31]–[34]. At the end of the 8-week treatment, the animals were placed in metabolic cages for 24 hrs urine collection. Proteinuria, albuminuria and creatinine were measured as previously reported [35]. A day prior to killing, the animals were weighed, anaesthetized with pentobarbital sodium (50 mg/kg i.p.), killed and the perirenal fat pads dissected free, blotted off water and weighed using an analytical balance (Precisa XR 205SM-DR, Precisa Instruments Ltd, Switzerland).

### HO-1 Concentration and HO Activity Assay

HO activity in the perirenal adipose tissue was measured as bilirubin production using our established method [24], [36], [37]. Briefly, the perirenal fat was homogenized on ice in 4 volumes of 5∶1 K/Na 100 mmol/L phosphate buffer with 2 mmol/L MgCl_2_ (HO-activity buffer), centrifuged at 13,000 rpm for 15 minutes. Aliquots of 100 µl were collected from the supernatant and transferred into another beaker containing 500 µl of a mixture of 0.8 mmol/L nicotinamide dinucleotide phosphate, 20 µmol/L hemin, 2 mmol/L glucose-6-phosphate, 0.002 U/µl glucose-6-phosphate dehydrogenase and 100 µl liver cytosol as source of biliverdin reductase. The reaction was done in darkness for 1 hour at 37°C, and was stopped by adding 500 µl of chloroform.

Thereafter, bilirubin was extracted by vigorously agitating the tubes and centrifuging at 13,000 rpm for 5 minutes, and the chloroform layer collected and read on a spectrophotometer at 464 nm minus the background at 530 nm. The amount of bilirubin in each sample was determined spectrophotometrically (extinction coefficient for bilirubin 40 mM^−1^cm^−1^), and expressed as nmole/mg protein/hour. The protein content was measured using Bradford assay. As a positive control, spleen tissue was used.

Perirenal fat HO-1 concentration was determined by enzyme-linked immunosorbent assay (ELISA) (EKS-810A, Stressgen-Assay Design, Ann Arbor, MI, USA) according to the manufacturer’s instructions as we previously reported [34], [38], [39].

### Histological, Morphological and Immunohistochemical Analyses of Kidney Tissue

Histology and morphometric analyses were done as we previously described [40]. Whole kidney sections of 5 µm were cut and treated with Masson’s Trichrome staining to assess collagen deposition. Morphologic assessment of collagen deposition was determined by a blinded researcher using a virtual microscope (Aperio Scan Scope Model CS, Aperio Technology Inc, CA), and analyzed using Aperio Image Scope V11.2.0.780 software (Aperio, e-Pathology Solution, CA). Each kidney section was magnified at 200X, and 20 random snaps were taken per slide per group of 4–6 animals (80–120 images per group), and subsequently scored semi-quantitatively by a blinded researcher as we previously reported [29], [40].

Immunohistochemistry was done as we previously reported [35]. Sections of 5 µm of whole kidney sections were treated with bovine serum albumin in phosphate buffered saline to block non-specific staining and incubated overnight with ED1 (1∶500 dilution, sc-59103, Santa Cruz Biotechnology, CA, USA) or HO-1 (1∶200 dilution, OSA-150, Stressgen Biotechnologies, Ann Arbor, MI, USA). Thereafter, the kidney sections were incubated with goat anti-mouse IgG for 30 min (1∶200 dilution; Jackson ImmunoResearch Laboratories, Inc., ME, USA). Immunohistochemical staining was performed using the standard avidin-biotin complex method with the chromagen 3,3′-diaminobenzidine (DAB) used at the final detection step. The kidney sections were scanned using a microscope (Aperio Scan Scope Model CS, Aperio Technology Inc, CA). Macrophages (brown from immune-stained sections) were quantified by manually counting the positively stained cells under a standard light microscope under 200X magnification in 20–22 randomized non-overlapping fields in the cortical region of kidney section, macrophages were infiltrated between intertubular spaces, in the glomeruli and perivascular region.

### Western Immunoblotting

The kidney was homogenized (1∶10, w:v) in 10 mM Tris-buffered saline (20 mM Tris-HCl, pH 7.4, 0.25 M sucrose, and 1 mM EDTA) in the presence of freshly prepared cocktail of protease inhibitors, centrifuged, and proteins extracted as we previously described [36], [41]. The proteins were extracted and quantified by Bradford assay, and aliquots of 50 µg were loaded on SDS-polyacrylamide gel.

The fractionated proteins were electrophoretically transferred to nitrocellulose paper and non-specific bindings blocked with 3% non-fat milk, and incubated overnight with primary antibodies against ED1, ED2, CD206, IL-10, nephrin, collagen-IV, and fibronectin (Santa Cruz Biotechnology, CA, USA). Anti-mouse glucose-6-phosphate dehydrogenase (G6PDH) antibody (Sigma St Louis, MO, USA) was used as control [42], [43] to ascertain equivalent loading. After washing, blots were incubated with anti-rabbit IgG conjugated to horseradish peroxide (Bio-Rad, CA, USA), and the immuno-reactivity visualized using enhanced horseradish peroxide/luminol chemiluminescence reagent (Perkin Elmer Life Sciences, Boston, MA, USA). Densitometric analysis was done with UN-SCAN-IT software (Silk Scientific, Utah, USA).

### Determination of Endothelin-1

Perirenal fat ET-1 was determined by EIA (Cayman Chemical, Ann Arbor, MI, USA) as we previously reported [38], [39]. This immunometric assay is based on a double-antibody ‘sandwich’ technique that detects ET-1 within the range of 0–250 pg/ml.

In brief, supernatants from homogenized perirenal fat tissues were purified by cold spike extraction, concentrated and the absorbance read at 405 nm in a plate reader (SpectraMax 340PC, Molecular Device, CA, USA) as we previously reported [29].

### Determination of TNF-α, IL-6 and IL-1β

Perirenal fat TNF-α, IL-6 and IL-1β were assessed by ELISA (Immuno-Biological Laboratories Co Ltd, Takasaki-shi, Gunma, Japan) according to the manufacturer’s instructions and read at 450 nm in a plate reader (SpectraMax 340PC, Molecular Device, CA, USA) as we previously reported [29].

### Determination of 8-isoprostane

8-isoprostane is a non-invasive index of oxidative stress. This was determined by EIA (Cayman Chemical, Ann Arbor, MI) as we previously reported [37]. The tissues were homogenized in phosphate buffer containing 0.005% butylated hydroxy toluene in a ratio of 10 µL buffer/mg tissue. Subsequently, an equal volume of 15% KOH was added to the homogenate. The samples were incubated at 40°C for an hour, followed by centrifugation, and the supernatant neutralized with KH_2_PO4 and the absorbance read at a wavelength of 412 nm in a microplate reader (SpectraMax 340PC, Molecular Device, CA, USA) and expressed as picograms per milligram of protein.

### Determination of Atrial Natriuretic Peptide (ANP)

Perirenal fat ANP was quantified by EIA (Cayman Chemical, Ann Arbor, MI, USA) as we previously reported [38]. This assay is based on the competition between unlabelled rat ANP and a tracer, acetylcholinesterase, linked to rat ANP for limited specific rabbit anti-rat ANP antiserum sites. The complex rabbit antiserum-rat ANP (free ANP or tracer) binds to the mouse monoclonal anti-rabbit antibody that is attached to a well. Briefly, supernatants from homogenized tissue were aliquoted wells containing unlabelled rat ANP and a tracer. After washing, Ellman’s Reagent (enzymatic substrate for acetylcholinesterase) and a chromogen added to the wells forming a yellow coloration that was read at 405 nm in a plate reader (SpectraMax 340PC, Molecular Device, CA, USA).

### Measurement of cGMP

The concentration of urinary GMP was measured by EIA (Cayman Chemical, Ann Arbor, MI, USA) as previously described [36], [44]. Urine samples were treated with 6% trichloroacetic acid at 4°C in the presence of 3′-isobutyl-1-methylxanthine to inhibit phosphodiesterase activity and centrifuged at 2000 g for 15 mins. The supernatant was recovered, washed three times with water-saturated diethyl ether and the upper ether layer was aspired and discarded while the aqueous layer containing cGMP was recovered and lyophilized. The dry extract was dissolved in 1 ml assay buffer and the cGMP content measured according to the manufacturer’s protocol and expressed as picomol per mg of protein.

### Determination of Macrophage-inflammatory-protein-1α (MIP-1α)

The concentration of MIP-1α was determined in homogenized perirenal and kidney tissues using ELISA kits (OmniKine™, Assay Biotechnology Company Inc, Sunnyvale, CA). All samples were assayed in triplicates following the manufacturer’s instructions.

### Determination of Plasma Adipopnectin

Adipopnectin was measured by ELISA (Phenix Pharmaceuticals, Inc, Burlingame, CA, USA) as we previously reported [23], [31]–[33]. In brief, plasma was aliquoted into wells of a microplate containing adiponectin antibody, followed by treatment with horseradish peroxidase-conjugated secondary antibody and streptavidin. Thereafter, the absorbance was read at 450 nm with a microplate (SpectraMax-340PC, Molecular Device, CA, USA).

### Statistical Analyses

All data are expressed as means ± SEM from at least four independent experiments unless otherwise stated. Statistical analyses were done using two-way ANOVA and Student’s *t-*test. Group differences at the level of p<0.05 were considered statistically significant.

## Results

### Hemin Therapy Abated Perirenal Adiposity and Reinstated Normoglycemia in ZDFs

The administration of hemin to ZDFs significantly reduced perirenal adiposity (**Table 1**). This was accompanied by a parallel reduction of hyperglycemia to normal levels (26.8±4.9 vs 7.1±1.5 mmol/L, p<0.01). In contrast, the co-administration of hemin and the HO-blocker, SnMP abolished the effect of hemin on perirenal adiposity and blood glucose, whereas treatment with SnMP alone aggravated perirenal adiposity and hyperglycemia suggesting a role of the HO system on the regulation of perirenal adiposity and glucose metabolism. The vehicle dissolving hemin and SnMP had no effect on blood glycemic and perirenal adiposity (**Table 1**).

**Table 1:** Effect of hemin and stannous mesoporphyrin (SnMP) on physiological variables in Zucker diabetic fatty (ZDF) and Zucker lean (ZL) rats.

Physiological variables	Animal groups							
	Control ZL	ZL+ Hemin	ZL+Vehicle	ControlZDF	ZDF+Hemin	ZDF+Hemin +SnMP	ZDF+SnMP	ZDF+Vehicle
**Body weight (g)**	366.4±7.5	357.8±6.4	361.5±4.9	389.5±8.6	371.5±5.7†	355.7±7.1§	363.9±8.1§	384.2±6.5
**Fasting glucose (mmo/L)**	7.0±0.2	6.2±0.3*	6.8±0.2	26.8±4.9	7.1±1.5**	23.5±5.1§	29.4±5.3§	25.8±4.2
**Perirenal adiposity**	10.5±2.7	8.2±1.2*	10.2±2.5	28.7±4.6	12.6±3.8**	26.4±5.6§	30.5±6.2§	29.5±5.3
**(g/Kg body weight)**								
**Albuminuria**	2.3±0.4	1.9±0.7	2.5±0.6	25.1±1.7	8.9±1.2**	27.1±2.8§	32.8±2.6§	26.7±3.9
**(mg/24 hrs)**								
**Proteinuria**	5.1±1.5	4.8±2.3	5.9±2.1	87.3±8.1	22.6±2.5**	90.7±7.8§	102.4±6.9§	91.5±10.6
**(mg/24 hrs)**								
**Creatinine Clearance (ml.min/g kidney)**	4.1±0.7	4.3±1.3	4.2±0.9	2.1±0.5	3.6±0.7**	2.4±0.5 §	1.8±0.4§	1.9±0.6

† p<0.05 vs controls;

* p<0.05,

** p<0.01 *vs* control ZDF or control ZL,

§ p<0.05 *vs* ZDF+Hemin, n = 6 per group.

Hemin therapy was also administered to ZL control rats. In hemin-treated ZLs, a slight but significant reduction of perirenal adiposity and blood glucose was observed (**Table 1**). In hemin-treated ZDFs, perirenal adiposity and glycemia were reduced by 56.1 and 73.5% respectively whereas in ZLs these same parameters were reduced by 21.9 and 11.4% respectively. These effects were abolished by the HO-blocker, SnMP. Interestingly, the effect of hemin was more-intense in unhealthy ZDFs than in the healthy ZLs, suggesting greater selectivity of the actions in ZDFs with disease.

Hemin and SnMP treatment caused a small reduction of body-weight (<9%) (**Table 1**). In ZL+hemin, ZDF+hemin, and ZDF+hemin+SnMP the loss of body-weight were 2.3, 4.6 and 8.7% respectively. Although body-weight loss may cause reduction of glycemia, it is unlikely in this case because the slight body-weight loss in hemin- and SnMP-treated animals were accompanied by opposite effects on glycemia; that is a reduction of glycemia in hemin-treated animals, but an increase in SnMP-treated animals (**Table 1**), suggesting that the HO system may be endowed with intrinsic anti-diabetic effects. The loss of body-weight may not be due to toxicity since we recently showed that indices of toxicity such as plasma alanine aminotransferase, gamma-glutamyltransferase and aspartate aminotransferase and were within normal range [23].

In order to examine the role of the HO system on renal function, important indices of renal function including urinary proteins like proteinuria and albuminuria [34] were assayed. Our results indicate that the levels of proteinuria and albuminuria were significantly elevated in untreated ZDFs (**Table 1**). Interestingly, hemin therapy markedly abated proteinuria and albuminuria by 74 and 64% respectively, whereas co-treatment of hemin and SnMP abolished the effects of hemin, while treatment SnMP alone exacerbated the excretion of these urinary proteins (**Table 1**). Furthermore, hemin therapy enhanced creatinine clearance in ZDFs, and thus improved renal function, whereas the HO-inhibitor, SnMP, nullified the effects of hemin on creatinine clearance. The vehicle dissolving hemin and SnMP had no effect on any of the measured parameters measured in ZDFs and ZLs.

### Hemin Therapy Enhanced HO-1 and HO Activity in Perirenal Adipose Tissue of ZDFs

**Figure 1:**
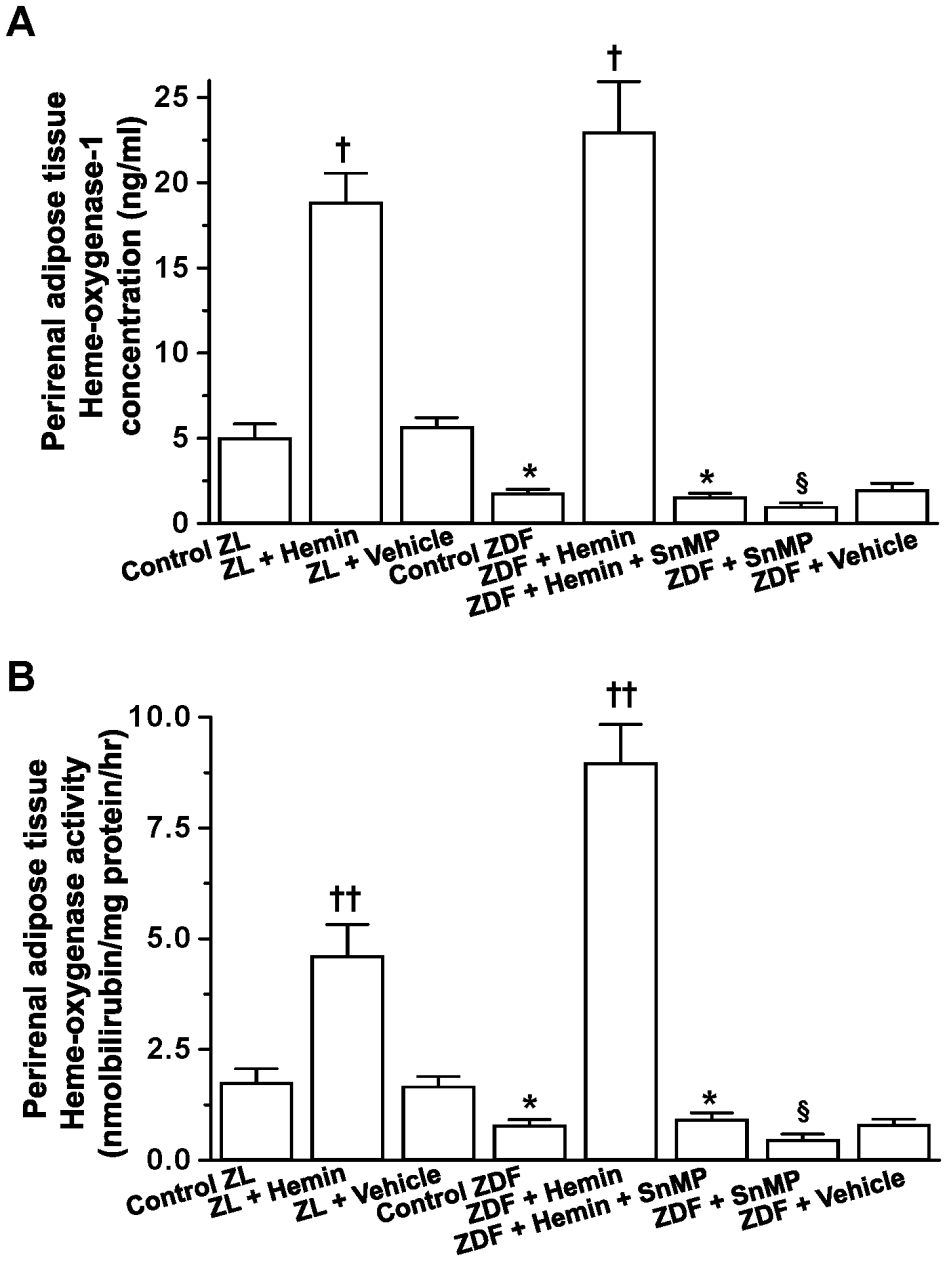
Effects of hemin, the HO inducer and SnMP, the HO inhibitor on HO-1 and HO activity of parirenal adipose tissue from ZDF and ZL rats. (**A**) The basal HO-1 levels in ZDF rats were lower than in age/sex-matched ZL-control rats, but were increased by hemin, whereas SnMP nullified the hemin effect. (**B**) The basal HO activity in ZDF rats was depressed as compared to ZL-control rats. Treatment with hemin markedly enhanced HO activity, whereas SnMP annulled the hemin effect. Hemin also enhanced HO-1 and HO activity in ZL rats, though less effectively as compared to ZDF rats. Bars represent means ± SEM; *n = 6* rats per group (*p<0.05 *vs* all groups, ^†^p<0.05, ^††^p<0.01 *vs* all groups;^ §^p<0.05 *vs* all groups).

To investigate the role of the HO system on perirenal adiposity and diabetic nephropathy, we measured HO-1 concentration and HO activity. Our results indicate that the basal levels of HO-1 and HO-activity in control-ZDFs were significantly reduced as compared to control-ZLs (**Figs. 1A and 1B** **)**. Interestingly, hemin therapy greatly enhanced the depressed levels of HO-1 and HO activity in ZDFs, whereas the co-treatment with the HO inhibitor, SnMP reversed the effects of the HO inducer, hemin, while treatment with SnMP alone depleted the basal levels of HO-1 and HO activity (**Figs. 1A and 1B**).

Hemin therapy also enhanced the levels of HO-1 and HO activity in ZL rats, although a greater increment was observed in hemin-treated ZDFs (**Figs. 1A and 1B**). In hemin-treated ZLs, HO-1 and HO-activity were increased by 3.8-and 2.6-fold respectively, while the increment observed in ZDFs for HO-1 and HO-activity were 13.5- and 11.2-fold respectively. The higher increment of HO-1 and HO-activity may account for the more accentuated effects against hyperglycaemia and nephropathy observed in ZDFs (**Table 1**). The vehicle dissolving hemin and SnMP had no effect on HO-1 and HO-activity in ZDFs and ZLs.

### Hemin Therapy Abated 8-isoprostane and ET-1 in Perirenal Adipose Tissue of ZDF

**Figure 2:**
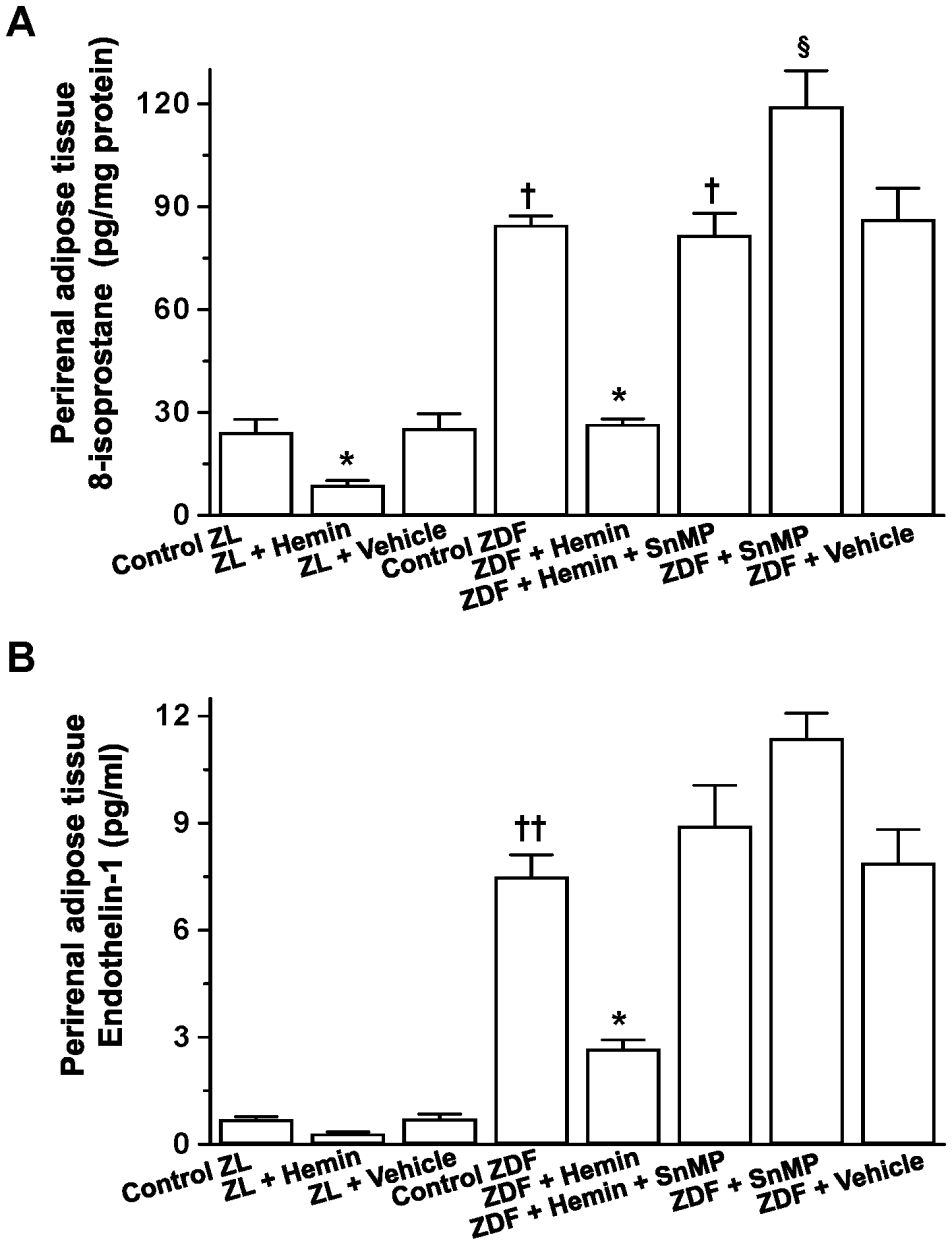
Effects of hemin, the HO inducer and SnMP, the HO inhibitor on 8-isoprostane and ET-1 of the parirenal adipose tissue from ZDF and ZL rats. (**A**) The basal 8-isoprostane levels in ZDF rats were markedly elevated as compared to ZL-control rats, but were significantly reduced by hemin, whereas SnMP nullified the hemin effect. (**B**) The basal ET-1 levels in ZDF rats were significantly elevated as compared to ZL-control rats, but were reduced by hemin, whereas SnMP nullified the hemin effect. Hemin also reduced 8-isoprostane and ET-1 in ZL rats, but less effectively as compared to ZDF rats. Bars represent means ± SEM; *n = 6* rats per group (*p<0.05 *vs* all groups, ^†^p<0.05, ^††^p<0.01 *vs* all groups;^ §^p<0.05 *vs* all groups).

Given that elevated oxidative stress is among the causative factors of insulin resistance and tissue dysfunction, we measured 8-isoprostane, an important marker of oxidative stress [45]. Moreover, ET-1 is implicated in cardiac and renal insufficiency [46]. In untreated ZDFs, the basal levels of perirenal adipose tissue 8-isoprostane were markedly elevated, suggesting enhanced oxidative stress (**Fig. 2A**). However, hemin therapy significantly reduced the elevated levels of 8-isoproatane by in ZDFs, whereas the co-treatment of hemin with SnMP nullified the effects of hemin while treatment with SnMP alone further increased 8-isoprostane levels, suggesting that oxidative stress is further potentiated by blockade of basal HO activity (**Fig. 2A**). Hemin therapy also reduced 8-isoprostane in ZL rats, although less-intensely as compared to ZDFs. In hemin-treated ZLs, 8-isoprostane was reduced by 2.7-fold as compared to a reduction of 3.3-fold in hemin-treated ZDFs (**Fig. 2A**).

Since 8-isoprostane stimulates ET-1 [47], and both ET-1 and 8-isoprostane are involved in the oxidative destruction of tissue, we also measured ET-1 in perirenal adipose tissue. Our results indicate that the levels of ET-1 in untreated ZDFs were significantly elevated as compared to the control-ZLs (**Fig. 2B**). Interestingly, hemin therapy significantly abated the elevated levels of ET-1 in ZDFs, while co-treatment of hemin and SnMP annulled the effect of hemin (**Fig. 2B**), whereas, treatment with SnMP alone further accentuated the levels of ET-1. Hemin therapy also reduced ET-1 in ZLs although to a lesser magnitude as compared to ZDFs. Accordingly, a reduction of 2.2-fold of ET-1 was observed in hemin-treated ZLs as compared to 2.8% hemin-treated ZDFs. The vehicle dissolving hemin and SnMP had no effect on 8-isoprostane and ET-1 in ZDFs and ZLs.

### Hemin Therapy Suppressed Pro-inflammatory Cytokines in Perirenal Adipose Tissue

**Figure 3:**
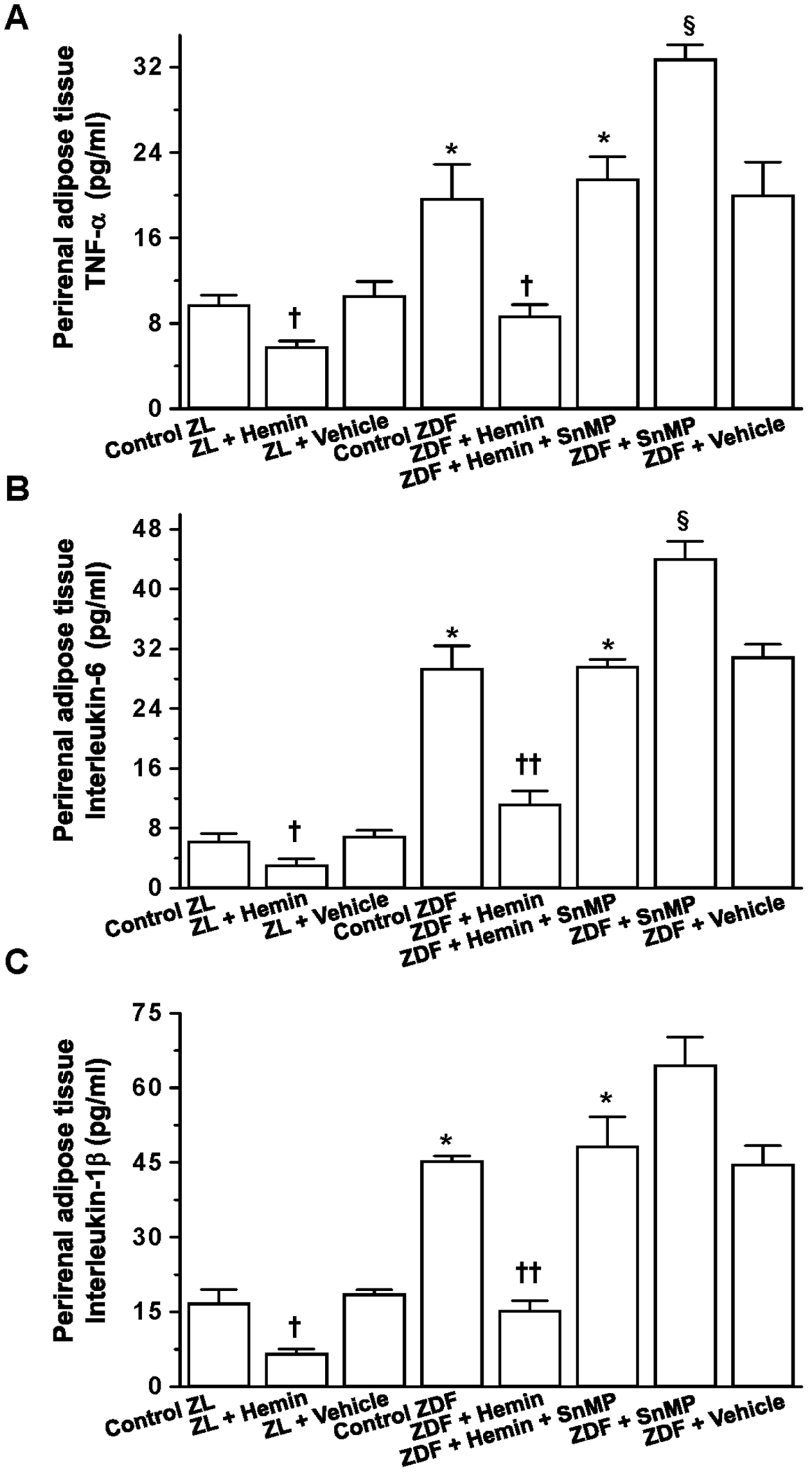
Effects of hemin, the HO inducer and SnMP, the HO inhibitor on TNF-α, IL-6 and IL-1β, of the parirenal adipose tissue from ZDF and ZL rats. Hemin therapy significantly reduced the elevated levels of (**A**) TNF-α, (**B**) IL-6 and (**C**) IL-1β in ZDF rats, but the hemin effects were reversed by co-treatment with the HO blocker SnMP, while treatment with SnMP alone further increased the levels. Hemin also reduced TNF-α, IL-6 and IL-1β in ZL rats, but less effectively as compared to ZDF rats. Bars represent means ± SEM; *n = 6* rats per group (*p<0.05, **p<0.01 *vs* all groups;^ †^p<0.05, ^††^p<0.01 *vs* all groups; **^§^**p<0.05, **^§§^**p<0.01 *vs* all groups).

TNF-α, IL-6 and IL-1β are cytokines that impair renal function and glucose metabolism [12]–[15], so we investigated whether the improvement of renal function and glucose metabolism in hemin-treated ZDFs would be accompanied by reduction of these cytokines. Our results indicate that the levels of TNF-α, IL-6 and IL-1β in perirenal adipose tissue of untreated ZDFs were significantly elevated as compared to control-ZLs (**Fig. 3A, 3B and 3C**). Treatment with hemin greatly reduced TNF-α, IL-6 and IL-1β, whereas the co-application of the HO-inhibitor, SnMP with hemin reversed the effects of hemin, while treatment of SnMP alone further increased the levels of these cytokines. Hemin therapy also reduced the levels of TNF-α, IL-6 and IL-1β in the ZLs, although less intensely. A reduction of 1.7-, 1.9-, and 2.5-fold of TNF-α, IL-6 and IL-1β respectively was observed in hemin-treated ZLs as compared to 2.2-, 2.6-, and 3.0-fold in hemin-treated ZDFs. The vehicle dissolving hemin and SnMP had no effect on TNF-α, IL-6 and IL-1β in ZDFs and ZLs.

### Hemin Therapy Potentiated ANP, its Surrogate Marker, Urinary cGMP and Adiponectin

**Figure 4:**
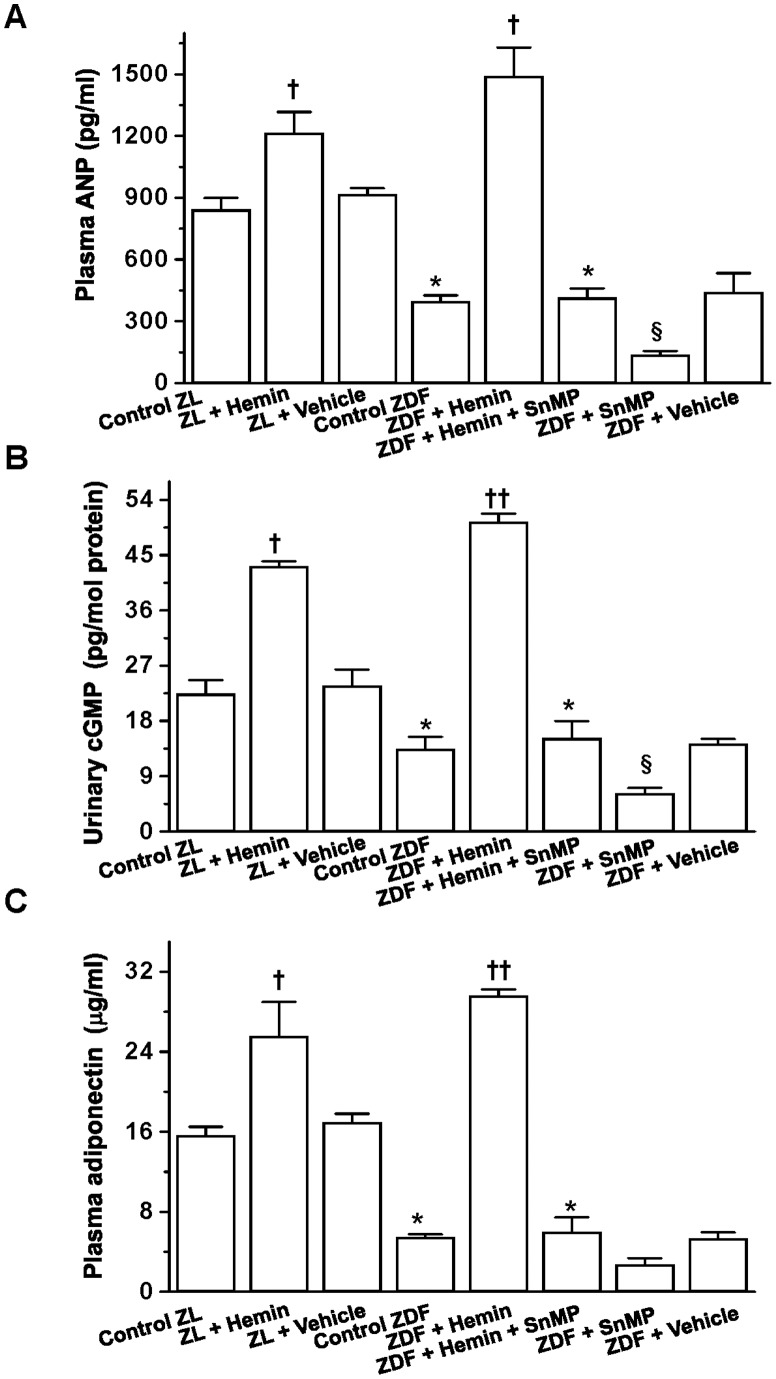
Effect of hemin, the HO inducer and SnMP, the HO inhibitor on plasma ANP, urinary cGMP and plasma adiponectin in ZDFs and ZLs. Hemin therapy significantly increased the depressed basal levels of (**A**) ANP, (**B**) urinary cGMP and (**C**) plasma adiponectin in ZDF rats, but was reversed by co-treatment with the HO blocker SnMP, while treatment with SnMP alone further depleted the basal levels. Hemin also increased plasma ANP, urinary cGMP and plasma adiponectin in ZL rats. Bars represent means ± SEM; *n = 6* rats per group (*p<0.05 *vs* all groups, ^†^p<0.01 *vs* all groups, ^§^p<0.05 *vs* all groups).

Given that ET-1 and ANP interact reciprocally [48], we investigated whether the hemin-induced reduction of ET-1 will affect the levels of ANP. Our results indicate that the reduction of ET-1 (**Fig. 2B**) by hemin was accompanied by the concomitant potentiation of ANP levels (**Figs. 4A** **)**. Consistently, urinary cGMP, a surrogate marker of ANP [49] was significantly increased by hemin, while co-treatment with the HO-inhibitor, SnMP reversed the effects of hemin (**Fig. 4B**), whereas, treatment with SnMP alone further depleted the basal levels of urinary cGMP.

Since adiponectin is an anti-inflammatory protein [50] with renoprotective effects and insulin sensitizing effects [51], [52], we investigated the effects of hemin on adiponectin in ZDFs. In ZDFs, the basal levels of adiponectin were depressed (**Fig. 4C**). Interestingly, hemin therapy significantly enhanced adiponectin (**Fig. 4A**). In contrast, co-treatment of hemin with the HO-inhibitor, SnMP annulled the hemin effects on adiponectin, while treatment with SnMP alone further reduced the levels of adiponectin. The vehicle dissolving hemin and SnMP had no effect on ANP and urinary cGMP.

### Hemin Therapy Abated MIP-1α in Perirenal Adipose Tissue and Kidneys

**Figure 5:**
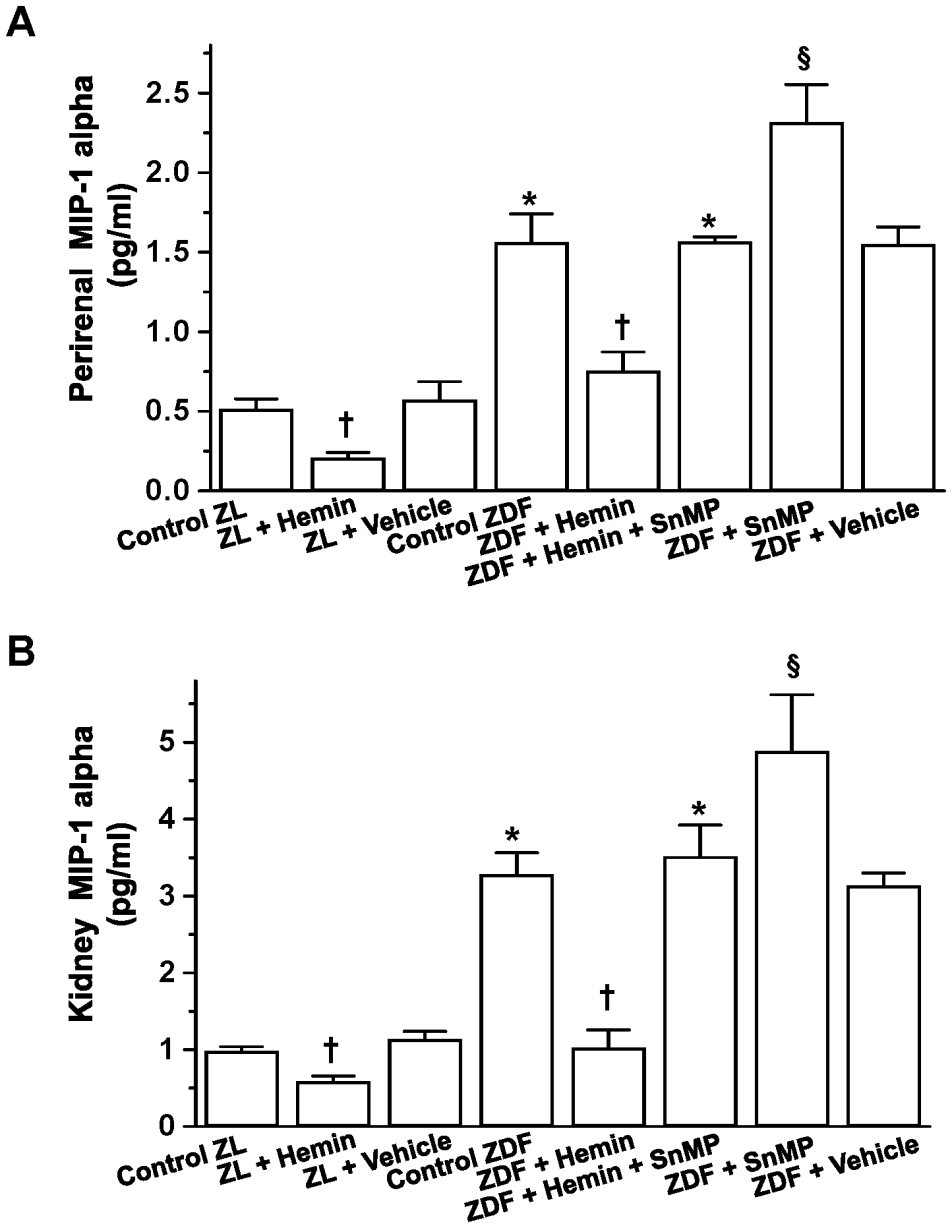
Effect of hemin on macrophage-inflammatory-protein-1 alpha (MIP-1α) in perirenal adipose tissue and the kidneys of ZDF. Hemin therapy significantly reduced the elevated levels of MIP-1α in (**A**) perirenal adipose tissues and (**B**) kidney from ZDF, but the hemin-effect was annulled by co-treatment with the HO blocker SnMP, while treatment with SnMP alone further increased the levels Bars represent means ± SEM; *n = 6* rats per group (*p<0.01 *vs* all groups; ^†^p<0.05, *vs* all groups; **^§^**p<0.01 *vs* all groups).

To further evaluate the effects of the HO system on macrophage infiltration, we determined the levels of MIP-1α, a chemokine implicated in macrophage infiltration [28]. The basal levels of MIP-1α in perirenal adipose tissue from control ZDFs were significantly elevated as compared to the ZL controls (**Fig. 5A**). Interestingly, treatment with hemin greatly attenuated the high levels of MIP-1α in ZDFs although comparable levels in ZL-controls were not reinstated. In contrast, the co-application of the HO-inducer, hemin with the HO-blocker, SnMP abolished the effects of hemin (**Fig. 5A**). Similarly, treatment with the SnMP alone exacerbated the levels of perirenal MIP-1α (**Fig. 5A**).

We also investigated the effects of hemin on kidney MIP-1α levels. In kidney tissues from control ZDFs, MIP-1α was markedly elevated as compared to ZL control rats (**Fig. 5B**), but was significantly attenuated by hemin therapy. On the other hand, the co-administration of SnMP and hemin nullified the effects of hemin (**Fig. 5B**), while SnMP alone further increased the levels of MIP-1α in ZDFs. It is noteworthy that hemin appeared to be more effective in the abrogating kidney MIP-1α as the hemin-dependent reduction of MIP-1α reinstated comparable levels as observed in ZL control rats. The reasons for this selective effect remain unclear, although tissue selectivity might be implicated.

Hemin therapy also reduced MIP-1α in perirenal adipose tissue and the kidney from ZL control rats (**Figs. 5A and 5B**). The vehicle dissolving hemin and SnMP had no effect on MIP-1α in ZDFs and ZLs.

### Hemin Therapy Abated Inflammatory Proteins Implicated in Insulin Resistance and Renal Dysfunction

**Figure 6:**
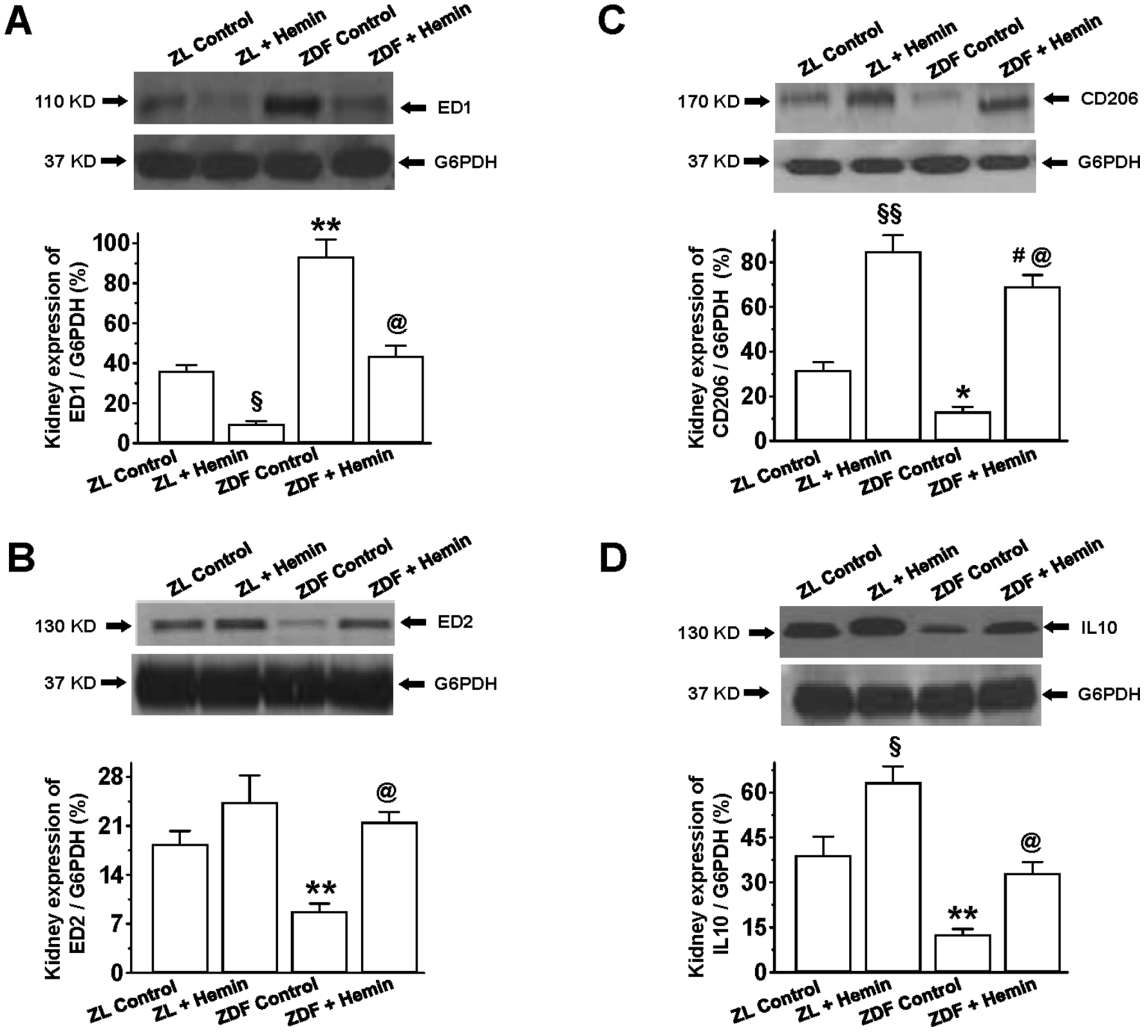
Effect of hemin on the expression of ED-1, ED2, CD206 and IL10 in renal tissues of ZDF. Representative Western immunoblots and relative densitometry indicates that hemin therapy significantly (**A**) reduced ED-1, but (**B**) enhanced ED2, (**C**) increased CD206, and (**D**) enhanced IL10 expression in ZDF. Bars represent means ± SEM; *n = 4* rats per group (*p<0.05, **p<0.01 vs ZL-Control; **^§^**p<0.05, **^§§^**p<0.01 *vs* ZL-Control; ^#^p<0.01 *vs* ZL-Control; ^@^p<0.01 *vs* ZDF-Control).

Given that macrophage infiltration is implicated in the development of insulin-resistant T2D and kidney dysfunction [13], [53]–[56], we used specific markers such as ED1 to quantify the pro-inflammatory M1-phenotype, and ED2, CD206 and IL10 for the assessment of anti-inflammatory M2-phenotype [27], [56]–[58]. Our Western immunoblotting and relative densitometric analyses revealed that the basal expression of ED1 in the kidneys of ZDF-controls were significantly elevated (**Fig. 6A**). Interestingly hemin therapy significantly attenuated the elevated expression of the pro-inflammatory M1-phenotype marker ED1 and restored ED1 to comparable levels as observed in ZL-controls (**Fig. 6A**). To determine whether the suppression of the pro-inflammatory M1-phenotype by hemin would be accompanied by changes in the anti-inflammatory M2-phenotype, we determined the expression of macrophage-M2 using specific M2 markers such as ED2, CD206 and IL10. Our results indicate that the basal expression levels of ED2, CD206 and IL10 were markedly reduced in ZDF-controls as compared to ZL-controls. Interestingly, hemin therapy robustly enhanced the depressed basal expressions of ED-2 (**Figs. 6B**), CD206 (**Figs. 6C**) and, IL10 (**Figs. 6D**), suggesting that hemin therapy may selectively modulate the polarization of macrophage toward the M2-phenotype that dampens inflammation. It is noteworthy that hemin therapy reinstated ED2 and IL10 to the levels of ZL-controls, but enhanced CD206 to levels even beyond ZL-controls. Hemin therapy also reduced ED1 in ZLs, and enhanced CD206 and IL10, but did not affect ED2 in ZLs.

### Hemin Therapy Suppresses Pro-fibrotic Proteins in the Kidney but Enhanced Nephrin

To further explore the mechanisms by which hemin therapy reduces proteinuria, and thus improve renal function, we assessed the expression levels of nephrin, an important transmembrane protein which forms the scaffolding of the podocyte slit diaphragm, a structure that regulates the aperture size of the renal filtration barrier, allowing the filtration of small molecules like ions, but not larger molecules like proteins [18]. A defect in nephrin causes massive excretion of proteins, hence proteinuria [18].

**Figure 7:**
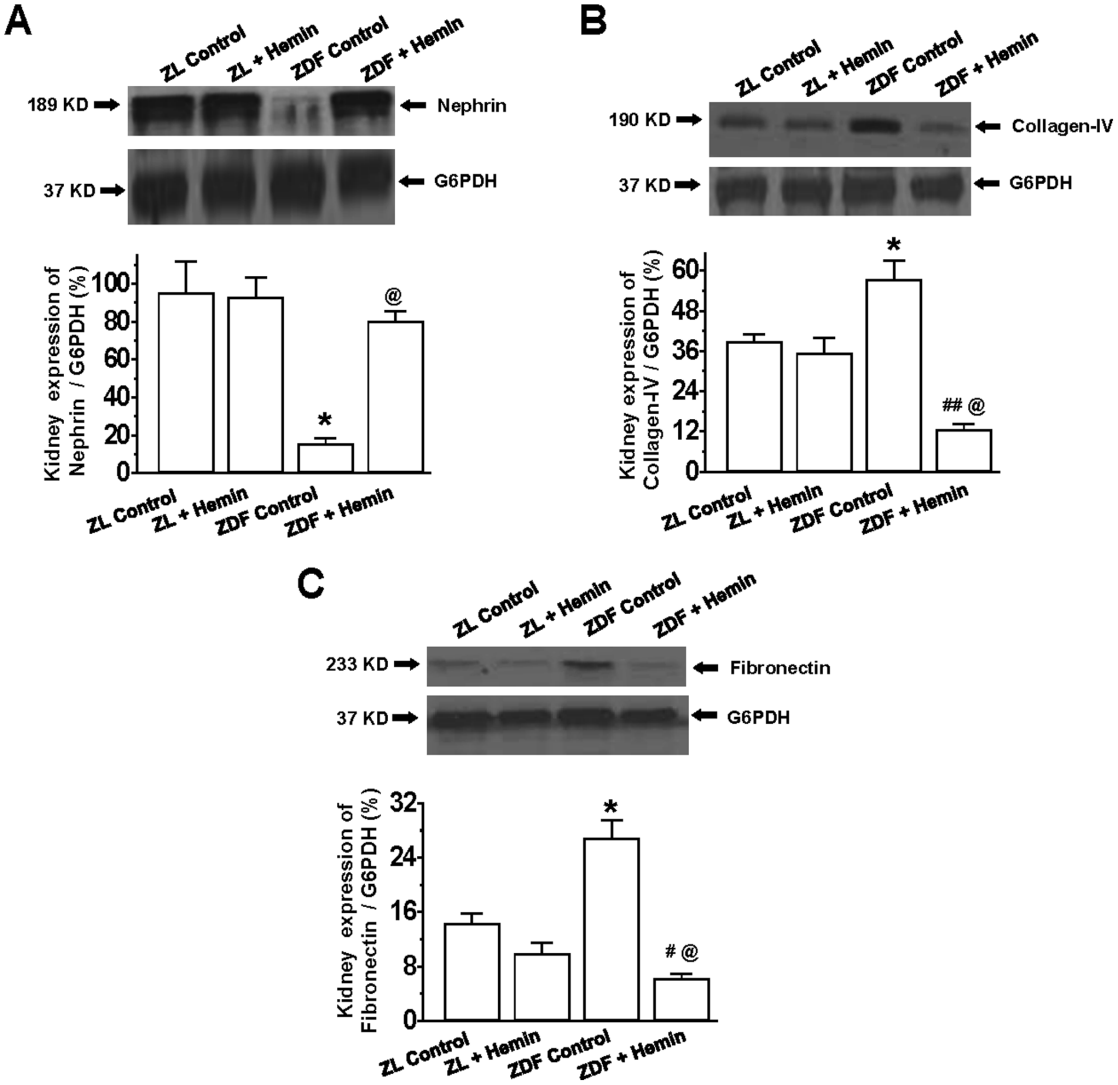
Effect of hemin on the expression of collagen-IV, fibronectin and nephrin in renal tissues of ZDF. Representative Western immunoblots and relative densitometry indicates that hemin therapy significantly (**A**) enhanced the expression of nephrin but, (**B**) abated collagen-IV expression, and (**C**) reduced the expression of fibronectin in ZDF. Bars represent means ± SEM; *n = 4* rats per group (*p<0.01 *vs* all groups; ^#^p<0.05, ^##^p<0.01 *vs* ZL-Control; ^@^p<0.01 *vs* ZDF-Control).

Our Western immunoblotting data indicates that in ZDF-controls, the basal expression of nephrin in the kidneys was markedly depressed as compared to the ZL-controls (**Fig. 7A**), and this coincided with marked increased in proteinuria and albuminuria, suggesting renal dysfunction (**Table 1**). However, treatment with hemin robustly enhanced the depressed expression of nephrin in ZDFs (**Fig. 7A**), reinstating comparable levels as observed in ZLs, with reduction of proteinuria and albuminuria (**Table 1**), and thus improved renal function. Hemin therapy did not affect the expression of nephrin in the healthy ZLs.

Since, the expression of nephrin is deregulated in diabetic nephropathy [18], [56], and elevated levels of pro-fibrotic/extracellular matrix proteins such as collagen and fibronectin are implicated in the aberrant expression of nephrin [19], [56], we investigated the effects of hemin therapy on the expression of collagen-IV and fibronectin. Our results indicate that in ZDFs, the basal expression levels of collagen-IV and fibronectin were significantly elevated as compared to the ZL-controls (**Figs. 7B and 7C**). Interestingly, the administration of hemin to ZDFs significantly reduced the elevated expression of collagen-IV and fibronectin to levels even lower than in the ZL-controls (**Figs. 7B and 7C**). Hemin therapy also reduced fibronectin expression in ZLs, but did not affect collagen-IV in ZLs. The reason for this selective effect is unknown, and should be further investigated. It is important to note that although hemin reduced fibronectin in healthy ZLs, it was more effective in unhealthy ZDFs because fibronectin was reduced by 4.3-fold in ZDFs as opposed to 1.5-fold in ZLs.

### Hemin Therapy Suppressed Renal Fibrosis

**Figure 8:**
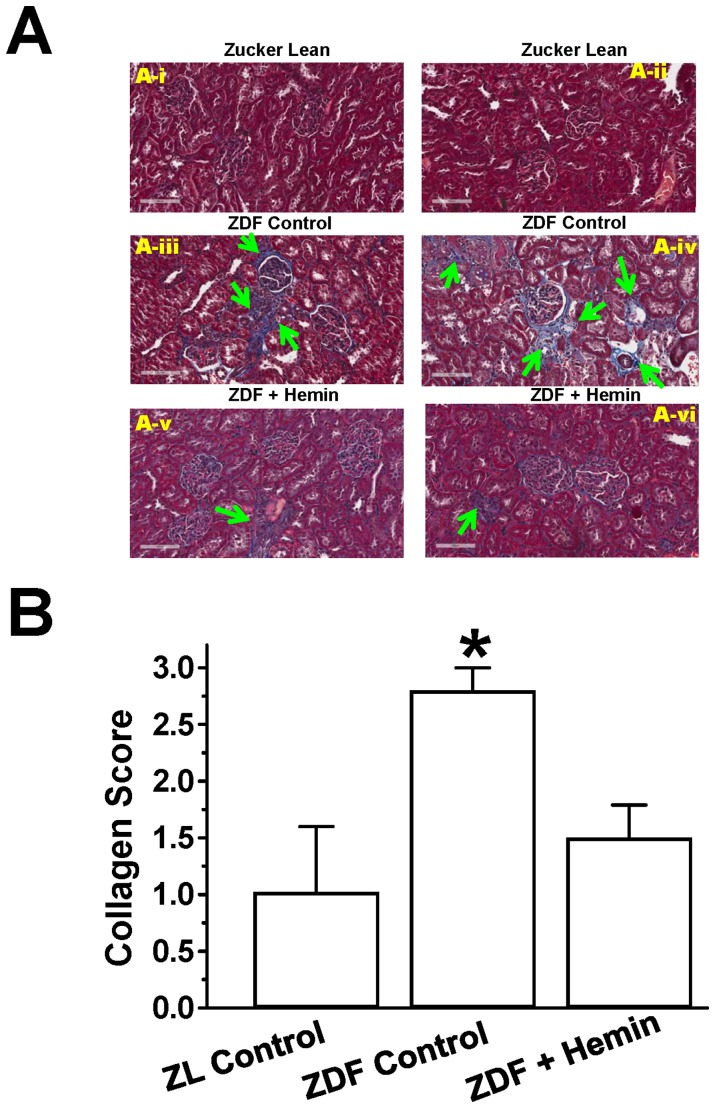
Masson’s trichrome staining of collagen deposition and fibrosis the in kidney. (**A**) Representative images of kidney section from two different rats. Sections from untreated ZDF-controls (panels ***A-iii*** and ***A-iv***) indicate severe fibrosis in tubulointerstitial, perivascular and glomerulus as compared with ZL-control rats (panels ***A-i*** and ***A-ii***), which interestingly were attenuated by hemin (panels ***A-v*** and ***A-vi***). (Magnification×200) (**B**) Semi-quantitative evaluation showed that hemin reduced collagen deposition. Bars represent means ± SEM; *n = 4–6* rats per group (*p<0.05 *vs* all groups).

Histological study using Masson’s trichrome staining and morphometric analyses were done to further confirm the renoprotective effects of hemin. As observed in (**Fig. 8A**), ZDF-controls displayed severe tubulo-interstitial, perivascular and glomerular fibrosis around the cortex and medullar as compared to ZL-controls. Similarly, kidney sections from ZDF-controls showed tubular vacuolization and glomerulosclerosis. Interestingly, these renal lesions were greatly attenuated by hemin therapy as hemin-treated ZDFs showed reduction of glomerular, tubulo-interstitial and perivascular fibrosis. Correspondingly, semi-quantitative analysis showed that hemin therapy significantly abated the elevated collagen deposition and perivascular fibrosis in ZDFs, reinstating similar levels as observed in ZL-controls (**Fig. 8B**).

### Hemin Therapy Suppressed Macrophage Infiltration in Renal Tissue by Abating ED1

**Figure 9:**
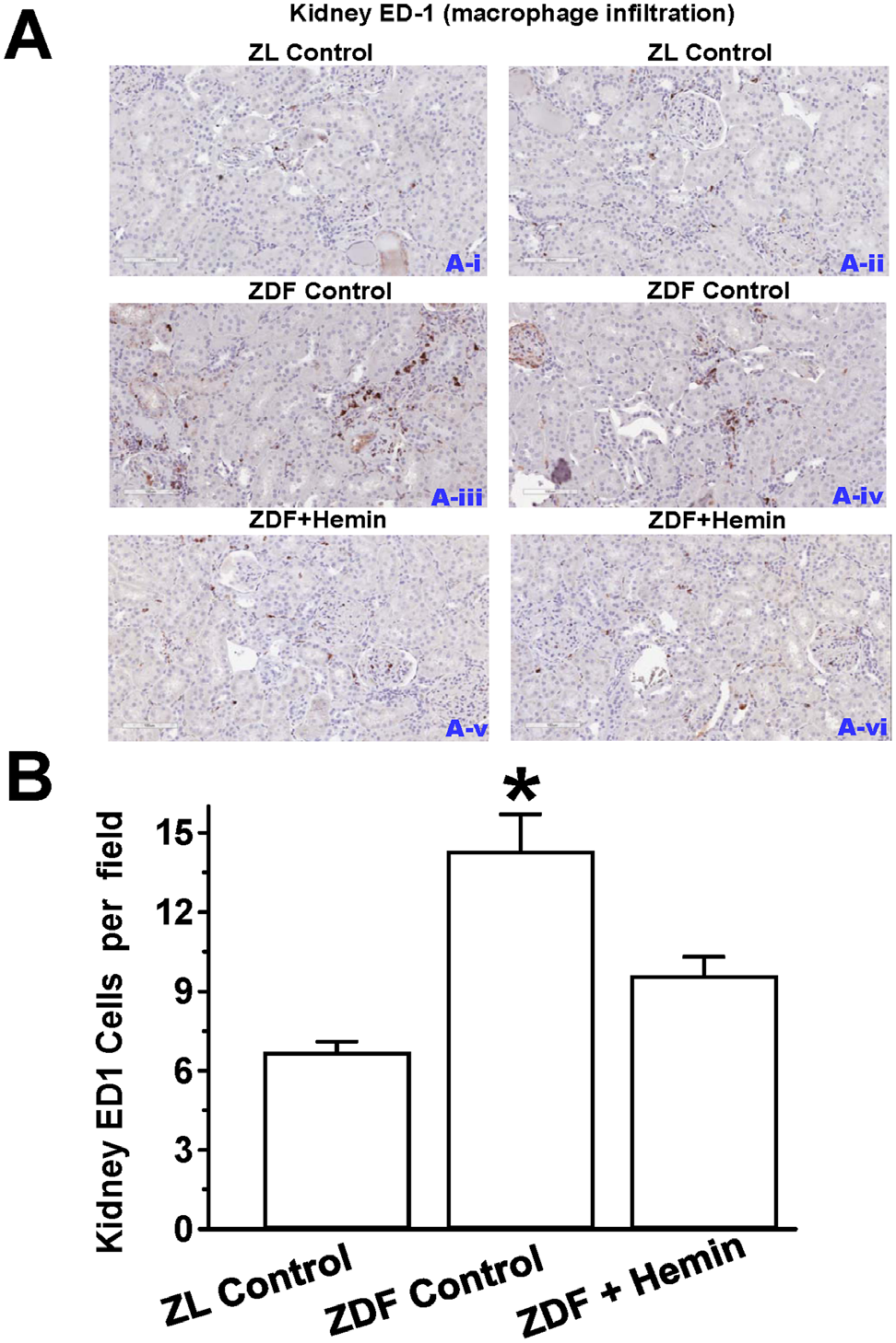
Effect of hemin therapy on kidney macrophage infiltration (A) Representative images of kidney section from different rats. The images reveal that macrophage infiltration (ED1-positive cells stained dark brown in kidney sections were elevated in ZDF-controls (panels ***A-iii*** and **A-iv**) as compared to ZL-controls (panels ***A-i*** and ***A-ii***), but interestingly were reduced by hemin (panels ***A-v*** and ***A-vi***). (Magnification×200). (**B**) Quantitative analyses per field indicating that in ZDF-controls macrophage infiltration was significantly elevated as compared to ZL-control, but was significantly attenuated by hemin therapy. Bars represent means ± SEM; *n = 4–6* rats per group (*p<0.01 *vs* all groups).

Since data from our Western immunoblot experiment indicated that hemin therapy abated ED1 expression in the kidney (**Fig. 6A**), we use the ED-1 antibody to determine macrophage infiltration in the kidneys by immunohistochemistry (**Fig. 9A**). Our results indicate that kidney sections from ZL-controls were almost devoid of the dark brown ED1 positive staining that characterizes macrophage infiltration. However, in untreated ZDF-controls, greater numbers of ED-1 positive staining for macrophage was observed in several structures located in the cortex and medullar of the kidney including the tubulointerstitial, perivascular and glomeruli as compared to ZL-controls (**Fig. 9A and 9B**). Interestingly, in hemin-treated ZDFs, there was a significant reduction in the number of ED-1 positively stained macrophage, suggesting reduction of macrophage infiltration. Correspondingly, hemin therapy significantly reduced the quantitative ED1 score of kidney sections (**Fig. 9B**), although the levels of ZL-controls were not reinstated.

### Immuno-labeling of HO-1 Shows Elevated HO-1 in Tubulointerstitial, Perivascular Area and Around the Glomeruli of Hemin-treated ZDF

**Figure 10:**
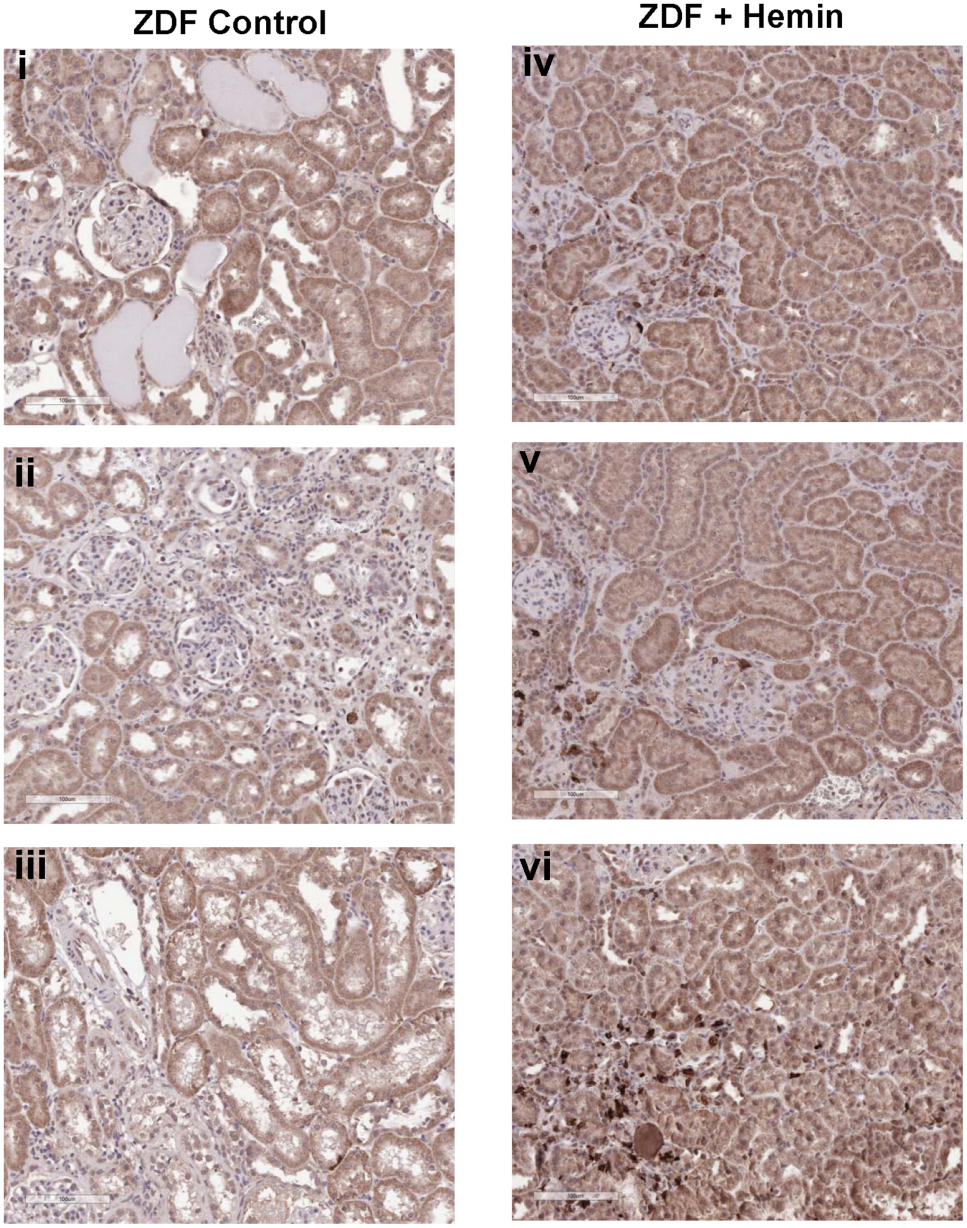
Immunolabelling of HO-1 in the kidney of ZDF-control and ZDF-treated with hemin therapy. Representative images of kidney section from different rats reveal that HO-1 is more expressed in ZDF+hemin group (panels ***iv–vi***) as compared to the ZDF-control group (panels ***i–iii***). (Magnification×200).

To further confirm the localization of HO-1 in the kidney, we did immunohistochemistry. Our immunohistochemical data shows very little expression of HO-1 was observed in kidney tissues of ZDF control (**Fig. 10**). However, in hemin-treated ZDF, HO-1 was very conspicuous and widely expressed in the renal parenchyma, with particularly high expressions in the tubulointerstitium, perivascular area and around the glomeruli.

## Discussion

The present study unveils several novel findings. These include **(i)** the hemin-induced enhancement of the anti-inflammatory macrophage M2-phenotype and corresponding reduction of the pro-inflammatory M1-phenotype; **(ii)** the suppression of perirenal adiposity and MIP-1α, a chemokine implicated in macrophage infiltration; (**iii**) the enhancement of nephrin, of nephrin, an important transmembrane protein critical for the formation of the podocyte slit diaphragm that regulates the aperture size of the glomerular filtration barrier, selectively allowing the filtration of small molecules like ions, but not larger molecules like proteins; and (**iv**) the corresponding reduction of proteinuria and albuminuria that was, interestingly, accompanied by increased creatinine clearance and thus improved renal function in ZDFs. The role of nephrin in glomerular filtration cannot be overemphasized. A defect in nephrin may cause massive excretion of proteins, hence proteinuria and renal dysfunction [20]–[22], [56]. It is possible that in ZDFs, the high levels of profibrotic/extracellular matrix proteins would aggravate histological renal lesions, and this defect was evidenced by increased tubular vacuolization, glomerulosclerosis with severe tubulo-interstitial, perivascular and glomerular fibrosis, all of which pathophysiological factors that together with the aberrant expression of nephrin may account for proteinuria and renal impairment. Therefore another important observation from our study is that hemin therapy significantly reduced the expression of pro-fibrotic/extracellular matrix proteins such as collagen and fibronectin. Moreover, excessive collagen and fibronectin are among the factors that deplete nephrin [17], a zipper-like protein that plays a fundamental role in the formation of the podocyte slit diaphragm of the glomerular barrier [18]–[20].

Although it is widely acknowledged that obesity and insulin resistant T2D are common causes of diabetic nephropathy and renal failure [3]–[5], [7], emerging evidence indicate that the anatomical location of adiposity reflects its adversity [10]. Therefore, the presence of excessive visceral adipose tissue like perirenal adiposity may constitute an independent prognostic factor of kidney malfunction in T2D [9]. Thus, the concomitant suppression of perirenal adiposity alongside the reduction of macrophage infiltration, the abrogation of mediators of oxidative stress like 8-isoprostane and ET-1 [45], [59], and the attenuation of pro-inflammatory cytokines like TNF-α, IL-6 and IL-1β [12]–[15] in perirenal adipose tissue by hemin are among the multifaceted mechanisms by which the HO system attenuate renal damage. In addition, the selective enhancement of anti-inflammatory M2-phenotype macrophage and corresponding reduction of the pro-inflammatory M1-phenotype in renal tissue from hemin-treated animals may be indicative of a novel mechanism by which the HO system counteracts tissue inflammation. In addition, it may also suggest a role of hemin therapy in the modulation of macrophage polarization.

During inflammation blood monocytes are recruited into the tissues where they differentiate into macrophages. Macrophage heterogeneity is a well-known phenomenon 13,25,26. Generally, macrophages heterogeneity reflects the specialization of tissue-resident macrophages in the different microenvironments in distinct tissues like liver, adipose tissue, kidney and other tissues 25,26. Within such microenvironment, macrophages can acquire distinct functional phenotypes 25,26. Importantly, macrophage polarization is driven by a wide variety of stimuli and signals in the tissue microenvironment, and these stimuli include cytokines, growth factors and other agents 25,26. The presence of these signals dictates the transcriptional response that shapes the phenotype and function of the macrophages on the basis of the physiological or pathophysiological role acquired by the macrophage in a given tissue 25,26. Therefore changes in the levels of the pro-inflammatory cytokines observed in hemin-treated animals may be responsible for the selective enhancement of the anti-inflammatory M2-phenotype. Moreover, during macrophage polarization, there is a switch of the gene expression program from a pro-inflammatory M1 signature to an anti-inflammatory M2-phenotype, depending on the tissue microenvironment and the presence of different stimuli including cytokines 25,26. Interestingly, hemin therapy suppressed the levels of several cytokines including TNF-α, IL-6 and IL-1β, and thus there is a possibility that in the microenvironment of the perirenal adipose tissue, the abrogation of these cytokines may account for the selective polarization of macrophage towards the anti-inflammatory M2-phenotype. Nevertheless, these preliminary observations made in this study are just the tip of an iceberg and more-intense research is needed to address the many challenging questions that would be necessary for characterizing the role of the HO system in macrophage polarization. Moreover, the suppression of macrophage infiltration and reduction of extracellular matrix/profibrotic protenis reported here are consistent with previous studied showing that upregulating the HO system is renoprotective 35,56,60.

Besides the selective enhancement of M2-phenotype, other mechanisms may account for the suppression of inflammation in hemin treated animals. These include the potentiation of ANP and the enhancement of adiponectin [50], [61], [62]. Interestingly, our results indicate that hemin therapy enhanced ANP and its surrogate marker, urinary cGMP [49], and adiponectin has been shown to enhance cGMP [63]. The stimulation of cGMP is an important mechanism by which ANP elicit its effects [64]. Given that impairment of cGMP-signalling leads to anti-thy1 glomerulonephritis [65], and the cGMP-signal transduction pathway has been shown to abate inflammation [66], the enhancement of ANP and cGMP by hemin may counter-regulate the effects of elevated renal inflammation to improve renal function. Thus, the cGMP secondary messenger system is a common denominator between the HO system, ANP and adiponectin. Therefore, the HO-adiponectin-ANP axis may constitute a synergistic protective axis with relevance for tissue defence and glucose metabolism. Moreover, hemin therapy may also abate inflammation and improve glucose metabolism by enhancing adiponectin, an anti-inflammatory protein with renoprotective and insulin sensitizing effects [50]–[52], [67]–[69]. Given that an ANP-mediated reduction of TNF-α, IL-6 and IL-1β have been linked to reduced insulin resistance [61], the suppression of these cytokines and potentiation of HO-adiponectin-ANP axis is important for enhanced glucose metabolism and improved kidney function observed in ZDFs. Therefore, the multifaceted mechanisms responsible for the renoprotection evoked by hemin include the potentiation of the HO system and related cellular targets like cGMP, ANP and adiponectin, which interestingly was accompanied by the reduction of collagen and fibronectin. The HO-adiponectin-ANP axis may suppress the adverse effects of ET-1. The interaction between ET-1 and ANP is well known [48]. For example ANP inhibits ET-1 [70], and interestingly, the hemin-induced increase of ANP was accompanied by a parallel reduction of ET-1. On the other hand, ANP attenuates fibrosis by abating extracellular matrix/profibrotic proteins including fibronectin and TGF-β1 [70], while ET-1 acts in concert with TGF-β1 to stimulate fibronectin synthesis [71]. Since ANP can also stimulate the production of adiponectin [72], a cytoprotective adipokine with anti-inflammatory effects [50], and the present study indicates that hemin therapy enhances adiponectin and ANP but abates ET-1 with the reduction of renal fibrosis, it could be envisaged that the potentiation of the HO-adiponectin-ANP axis is an important renoprotective mechanism.

Our study also indicates that the effect of hemin therapy was less-pronounced in ZL-control rats with healthy status, suggesting greater selectivity of the HO system in ZDFs with disease. Alternatively, the HO system in healthy ZL-control rats may be more stable given that HO-1 and HO-activity in ZDFs were depressed as compared to ZLs, and interestingly, the effect of hemin on HO-1 and HO-activity was more accentuated in ZDFs than ZLs. It is also possible that the higher magnitude of HO-signalling in hemin-treated ZDFs may be responsible for the more intense anti-diabetic and reno-protective effect in ZDFs as compared to ZLs. This is reflected in the physiological variables measured (Table 1). In hemin-treated ZDFs, fasting glucose, perirenal adiposity, proteinuria and albuminuria were reduced by 73.5, 56.1, 74.1 and 64.6% respectively, while creatinine clearance was increased by 41.7%. On the other hand, fasting glucose, perirenal adiposity, proteinuria and albuminuria were only reduced in hemin-treated ZLs by 11.4, 21.9, 5.9 and 17.4% respectively, with only a 4.7% increase in creatinine clearance. It is important to note that although these physiological variables in ZLs were affected by hemin, they were still within the acceptable physiological range. Similarly, most of the other biochemical parameters measured in this study were more accentuated in hemin-treated ZDFs than hemi-treated ZLs. Therefore the hemin-mediated changes in physiological variables and biochemical parameters reported in this study are more intense in unhealthy ZDFs than healthy ZLs, suggesting greater selectivity of hemin in diseased conditions. Nevertheless, further investigations needs to clarify the selectivity of hemin therapy in unhealthy ZDFs.

Immunohistochemical labeling of HO-1 in kidney has been widely reported [60], [73]–[75]. These studies indicate that HO-1 is expressed all over the renal parenchyma, with higher levels in tubular epithelial cells, vascular wall smooth muscle cells and the interstitium of the cortex and medulla [60], [73]–[75]. Consistently, our immunohistochemical results show high HO-1 expression in many areas of the renal parenchyma including the tubulointerstitium and perivascular area of the cortex and medullar. Interestingly, these areas intense HO-1 expression coincided with areas of the kidney where lesions were hemin therapy significantly reduced histopathological lesions. Moreover, in our study, renal lesions and kidney insufficiency was associated with severe histopathological lesions in tubular epithelial cells, the interstitium and other regions of the cortex and medullar in untreated ZDFs. These renal lesions were evidenced by increased tubular vacuolization, glomerulosclerosis with severe tubulo-interstitial, perivascular and glomerular fibrosis, and were associated with elevated macrophage infiltration and increased deposition of collagen, an extracellular matrix protein in untreated ZDFs. Moreover, the elevated collagen deposition in untreated ZDFs was associated with increased expression of fibronectin, another extracellular matrix protein that together with collagen are known to deplete nephrin causing proteinuria and renal insufficiency [19]–[22], [56]. Given that previous studies have reported increased HO-1 expression in the interstitium and tubular epithelium of the cortex following the administration of HO-inducers [60], [74], and incidentally the interstitium and tubular epithelium were among the areas with significant lesion in untreated ZDF, the present study and previous reports in literature strongly suggest that the HO system alleviates not only tubulo-interstitial injury, but also suppress perivascular and glomerular fibrosis to improve renal function.

Generally, HO-1 is activated by a wide variety of physical, chemical and pathophysiological stimuli [76]–[81]. Accordingly, HO-1 may be considered a sensitive index that is triggered during the onset of pathophysiological alterations in tissues as an attempt to counteract the adverse changes. However, the pathophysiological activation of HO-1 has been shown to evoke only a transient or sub-threshold value of HO-activity that is incapable of activating important downstream signaling components of the HO system like cGMP [36], [41], [56], [80], [82]–[85], suggesting the necessity for a more robust enhancement of HO-1 by pharmacological agents like cobalt proptoporphyrin and hemin [36], [41], [82]–[85]. Thus, the transient up-regulation of HO-1 that accompanies many pathophysiological conditions may represent the first line of defense mounted by the HO system against tissue injury. Accordingly, the high expression of HO-1 in interstitial macrophages and tubular epithelial cells reported in the cortex and outer medullar regions of dysfunctional kidneys [60], [73] may be indicative of the manifestation of pathophysiological alterations in the kidneys. Similarly in our study we observed renal insufficiency characterized by elevated proteinuria/albuminuria and reduced creatinine clearance in untreated ZDFs. However, these renal defects were attenuated by potentiating the HO system and its downstream signaling molecule cGMP by hemin therapy.

Collectively, the present findings indicates that the concomitant enhancement of ANP, cGMP, adiponectin and creatinine clearance, alongside the corresponding reduction of perirenal adiposity and the suppression of pro-inflammatory/oxidative mediators, macrophage infiltration, albuminuria and proteinuria may account for the improved glucose metabolism and improved renal function in hemin-treated ZDFs. These data suggest that HO-inducers like hemin may be explored against the co-morbidity of perirenal adiposity and diabetic nephropathy.

## Limitations

Although the present study underscores the renoprotective effects of hemin therapy in diabetic nephropathy, and suggest that the suppression of extracellular matrix proteins like collagen-IV and fibronectin in hemin-treated ZDF are accompanied by increased expression of nephrin and improved renal function, these observations should be cautiously interpreted because this study does not provide unequivocal data that demonstrate the interaction among HO-1, collagen-IV, fibronectin and nephrin in the glomeruli.
